# Effect of physical activity and sun exposure on vitamin D status of Saudi children and adolescents

**DOI:** 10.1186/1471-2431-12-92

**Published:** 2012-07-03

**Authors:** Abdulaziz Al-Othman, Sara Al-Musharaf, Nasser M Al-Daghri, Soundararajan Krishnaswamy, Deqa S Yusuf, Khalid M Alkharfy, Yousef Al-Saleh, Omar S Al-Attas, Majed S Alokail, Osama Moharram, Shaun Sabico, George P Chrousos

**Affiliations:** 1 Prince Mutaib Chair for Biomarkers of Osteoporosis, King Saud University, Riyadh, Kingdom of Saudi Arabia; 2 College of Applied Medical Sciences, King Saud University, Riyadh, Kingdom of Saudi Arabia; 3 College of Science, King Saud University Women's Section, Riyadh, Kingdom of Saudi Arabia; 4 Biomarkers Research Program, Biochemistry Department, College of Science, King Saud University, Riyadh, Kingdom of Saudi Arabia; 5 Center of Excellence in Biotechnology Research, King Saud University, Riyadh, Kingdom of Saudi Arabia; 6 Clinical Pharmacy Department, College of Pharmacy, King Saud University, Riyadh, Kingdom of Saudi Arabia; 7 College of Medicine, King Saud University of Health Sciences, Riyadh, Kingdom of Saudi Arabia; 8 King Abdulaziz University Hospital, King Saud University, Riyadh, Kingdom of Saudi Arabia; 9 First Department of Pediatrics, Athens University Medical School, Athens, 11527, Greece; 10 Biochemistry Department, College of Science, King Saud University, PO Box, 2455, Riyadh, 11451, Kingdom of Saudi Arabia

**Keywords:** Vitamin D, Saudi children

## Abstract

**Background:**

Accumulating evidence suggests an increased prevalence of vitamin D deficiency in the Middle East. In this context, we aimed to determine whether the prevalence of vitamin D deficiency is related to degree of physical activity and sun exposure among apparently healthy Saudi children and adolescents, a little studied population.

**Methods:**

A total of 331 Saudi children aged 6–17 years (153 boys and 178 girls) were included in this cross sectional study. Levels of physical activity and sun exposure were determined using a standard questionnaire. Anthropometry, serum calcium and 25-(OH) vitamin D were analyzed.

**Results:**

All subjects were vitamin D deficient, the majority being moderately deficient (71.6%). Age was the single most significant predictor affecting 25 (OH) Vitamin D levels, explaining 21% of the variance perceived (*p* = 1.68 x 10^-14^). Age-matched comparisons revealed that for groups having the same amount of sun exposure, those with moderate or are physically active will have higher levels of vitamin D status, though levels in across groups remained deficient.

**Conclusion:**

Vitamin D deficiency is common among Saudi children and adolescents, and is influenced by both sun exposure and physical activity. Promotion of an active outdoor lifestyle among Saudi children in both homes and schools may counteract the vitamin D deficiency epidemic in this vulnerable population. Vitamin D supplementation is suggested in all groups, including those with the highest sun exposure and physical activity.

## Background

The prevalence of vitamin D deficiency has increased rapidly worldwide in both children and adults during the past decade [1-3]. Around 30 to 50% of children and adults in the United Arab Emirates, Australia, Turkey, India, and Lebanon have 25(OH)D levels below 20 ng/ml [4-7]. The high prevalence of vitamin D deficiency may be due to low exposure of the skin to ultra violet radiation (UVB) [8], higher skin pigmentation and low intake of vitamin D [9]. Other factors such as mutations in vitamin D receptor (VDR) polymorphism [10], low daily calcium intake [9], obesity and low social status have all been associated with low circulating vitamin D levels [11,12].

The kingdom of Saudi Arabia (KSA) has unique conditions that influence vitamin D status even though Saudi Arabia is sun-drenched throughout the year and temperatures often rise above 50 °C (122 °F) during summer. A recent study revealed counterintuitive effects of a season to vitamin D status amongst Saudi locals and expatriates, revealing higher vitamin D status levels during winter months as compared with the summer [13]. Saudis usually limit the time they spend outdoors during the day time. In summer months, for example, parents do not allow their children to engage in outdoor activities during daytime. Women in particular receive little or no sun-light since they cover their bodies with dark veils completely, for cultural and religious reasons.

The aim of the present study was to determine the prevalence of vitamin D deficiency and its association with behavioral factors like physical activity and exposure to sun light in an otherwise healthy group of Saudi children, an understudied population in terms of vitamin D deficiency. The results should help in raising awareness as well as suggesting strategies to combat vitamin D deficiency in this part of the world and thus in reducing the incidence of various chronic diseases associated with vitamin D deficiency (e.g., osteomalacia, metabolic syndrome, heart diseases…).

## Methods

### Subjects

In this cross-sectional study, a total of 331 apparently healthy Saudi boys and girls aged 6–17 years were enrolled from 4 different Primary Health Care Centers in different areas within Riyadh city during the months of March-December 2010, majority of whom were recruited during the summer months of April to November. Written informed consents for parents as well as assent for children and/or adolescents were obtained prior to inclusion. Subjects with chronic conditions such as asthma, type 1 diabetes mellitus, hypertension, history of cardiac, kidney or liver disease, use of medications known to affect body weight (such as steroids), psychiatric conditions, and those taking calcium, vitamin D or multivitamin supplements were excluded from the study.

Ethical approval was obtained from the Ethics Committee of the College of Science Research Center, King Saud University, Riyadh, Saudi Arabia. A pre-designed and approved questionnaire used in a previous study [14] which includes medical history was answered for all participants with the help of their parents. The questionnaire also sought information about sun exposure (frequency of exposure, duration of exposure) and physical activity, which is self reported (frequency and type of activities performed along with duration – number of minutes per week).

### Anthropometric measurements

Subjects were assigned to visit the primary care most convenient for them for the collection of anthropometric data and blood extraction. Physical examination was carried out by the attending physician who ensured that the participants met the inclusion and exclusion criteria. Weight and height were recorded to the nearest 0.2 kg and 0.5 cm, respectively, using an appropriate international standard scale (Digital Pearson Scale, ADAM Equipment Inc., USA). Blood pressure was measured using an appropriate mercurial sphygmomanometer. Blood pressure was measured twice with 15-minute intervaland the mean of the two readings was recorded.

### Serum biochemical analysis

Blood (≈ 10 cc) was withdrawn by a nurse after an overnight fast (> 10 hours) on the same day anthropometric information was gathered. Serum calcium was measured using standard analytical techniques (Konelab, Finland). Fasting blood glucose and lipids which included triglycerides, total, HDL- and LDL-cholesterol were measured routinely using the a chemical analyzer (Konelab, Finland). 25 (OH) Vitamin D was measured using enzyme-linked immunosorbent assay (ELISA) (I.D.S., Tyne & Wear, UK). The inter- and intra-assay variability of this assay was 5.3 and 4.6%, respectively.

### Statistical analysis

Statistical analysis was carried out using the Statistical Package for the Social Science (SPSS 11.5, Chicago IL, USA). Frequencies were expressed in percentage (%), continuous variables were presented as mean ± standard deviation. Analysis of variance (ANOVA) was done to compare age-matched groups. Variables such as triglycerides and LDL-cholesterol were log transformed prior to parametric comparisons. Stepwise linear regression analysis was done to determine which among the parameters measured are significant predictors for 25(OH)D (dependent variable). P-value significant at p < 0.05.

## Results

### Prevalence of vitamin D deficiency

**Figure 1:**
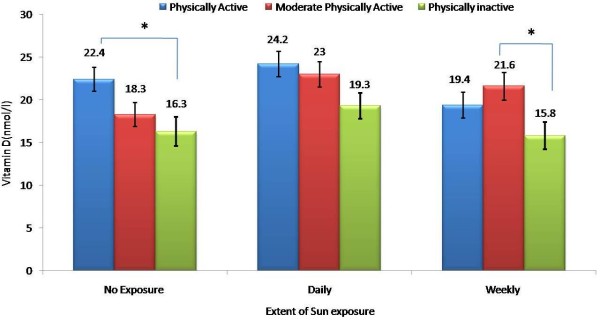
Effect of physical activity on 25(OH) vitamin D levels according to level of sun exposure; * denotes p-value < 0.05.

Based on their vitamin D deficiency status using different cut-off values: severe (<12.5 nmol/l), moderate (12.5-24.9 nmol/l), and mild (25.0-29.9 nmol/l) [15], 11.4% of subjects had severe vitamin D deficiency while 71.6% and 16.8% of subjects showed moderate and mild deficiencies, respectively (not shown in table). Age was the single most significant predictor affecting 25 (OH) Vitamin D levels, explaining 21% of the variance perceived (*p* = 1.68 x 10^-14^) Figure 1.

### Physical activity and vitamin D

**Figure 2:**
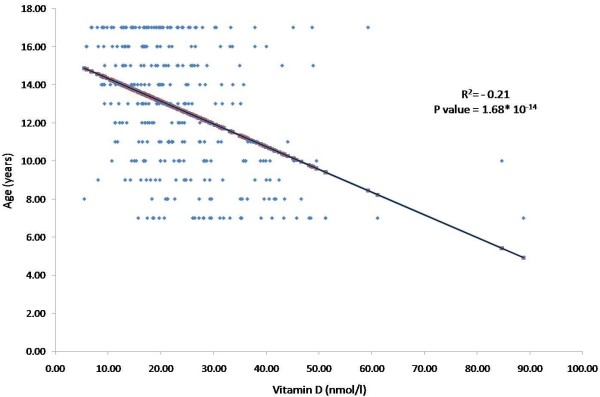
Linear regression between 25(OH) vitamin D and age.

The subjects were divided into three groups based on their level physical activity, subjects were divided into three groups: inactive, moderately active and active. The subjects who were physically inactive were approximately 15% in both males and females, while the physically active subjects were 27% and 17% in males and females, respectively. The lowest mean level of 25(OH)vitamin D in serum was found in the physically inactive group [17.7 ± 1.6 nmol/l vs 22.7 ± 1.5 nmol/l (*p* < 0.05)]. The BMI was highest in the physically inactive group and this was borderline significant [23.1 ±7.9 vs 20.1 ± 5.8 (p = 0.05)] (Table 1). Serum fasting glucose was also highest in the physically inactive group (*p* = 0.03). In all groups, levels of 25(OH)D were less in the group with lesser physical activity, but significance was only noted in the no exposure group (*p* = 0.04) as well as the moderate physically active group versus physically inactive group under the weekly exposed group (*p* = 0.03) (Figure 2).

**Table 1:** General Characteristics of Subjects Based on Level of Physical Activity

**Parameters**	**Physically Inactive**	**Moderate PA**	**Physically Active**	**P value**
N	52	205	74	
Boys/Girls	25/27	87/118	41/33	
Age (years)	13.4 ± 3.2	12.6 ± 3.3	12.1 ± 3.6	0.11
BMI (kg/m^2^)	23.1 ± 7.9	21.6 ± 6.6	19.9 ± 5.8*	0.05
Waist (cm)	67.8 ± 26.2	66.6 ± 19.4	63.8 ± 17.8	0.51
Hips (cm)	82.8 ± 29.1	82.2 ± 21.3	78.4 ± 20.6	0.43
WHR	0.82 ± 0.10	0.81 ± 0.11	0.81 ± 0.08	0.85
Systolic BP (mmHg)	110.9 ± 10.7	108.2 ± 11.6	109.2 ± 11.8	0.36
Diastolic BP (mmHg)	70.9 ± 12.8	70.9 ± 10.4	71.2 ± 9.7	0.97
Glucose (mmol/L)	5.3 ± 0.91	5.2 ± 0.64	4.9 ± 0.81*	0.03
Triglycerides (mmol/l)#	0.97 ± 0.53	0.91 ± 0.47	1.0 ± 0.69	0.42
Total Cholesterol(mmol/l)	4.2 ± 0.58	4.0 ± 0.67	4.2 ± 0.90	0.31
HDL Cholesterol (mmol/l)	1.0 ± 0.38	1.0 ± 0.27	1.0 ± 0.29	0.73
LDL Cholesterol (mmol/l)#	2.8 ± 0.71	2.7 ± 0.65	2.7 ± 0.53	0.71
25(OH) Vitamin D (nmol/l)	17.7 ± 1.6	21.2 ± 1.6*	22.7 ± 1.5*	0.01

Data presented as mean ± standard deviation; * denotes significance as compared to physically inactive; # denotes non-Gaussian variables. Significance at p < 0.05.

### Sun exposure and vitamin D

Associations of 25 (OH) vitamin D were determined in subjects according to 3 groups based on level of exposure to sunlight. These were, no exposure (0 minute), daily exposure (10–30 minutes) and weekly exposure (40–160 minutes). Around 24% of the subjects had no exposure to sun light, 39.5% had once in a week exposure and 36.5% had daily exposure. Serum vitamin D levels increased with increasing sun exposure (not shown in table). Age was negatively and significantly associated with 25 (OH) vitamin D levels in all groups as well as BMI and hips in the no exposure and weekly exposure groups (Table 2).

**Table 2:** Bivariate Associations of 25(OH) Vitamin D to Various Parameters According to Sun Exposure

**Parameters**	**No exposure**	**Daily exposure**	**Weekly exposure**
Age (years)	- 0.59**	- 0.36**	- 0.40**
BMI (kg/m^2^)	- 0.25*	- 0.18	- 0.19*
Waist (cm)	- 0.16	- 0.12	- 0.18
Hips (cm)	- 0.25*	- 0.20	- 0.25*
WHR	0.31	0.17	0.12
Systolic BP (mmHg)	0.01	- 0.15	0.06
Diastolic BP (mmHg)	- 0.004	- 0.07	0.07
Glucose (mmol/L)	- 0.04	- 0.07	0.07
Triglycerides (mmol/l)	- 0.12	- 0.14	- 0.05
Total Cholesterol(mmol/l)	0.01	- 0.17	- 0.07
HDL Cholesterol (mmol/l)	0.05	- 0.12	- 0.08
LDL Cholesterol (mmol/l)	- 0.23	- 0.05	- 0.04

Data presented as coefficient (R); * denotes significance at < 0.05; ** denotes significance at < 0.01.

## Discussion

The important findings of this study reveal that vitamin D deficiency in children and adolescents is very common in Saudi Arabia as noted previously and elsewhere in the Middle East [16-18], and is associated with lack of physical activity and sun exposure. Daytime outdoor physical activity may act as a surrogate indicator for sun exposure, however, exercise in itself may contribute to the maintenance of vitamin D status, other than merely by increasing exposure of skin to sunlight [19]. Consistent with our findings, Brock et al. reported that the major modifiable predictors of low vitamin D status were body mass index (BMI) >30 kg/m^2^, physical inactivity and calcium supplement intake [20]. It is well established that physical activity increases local bone mass, reduces calcium excretion and raises absorption efficiency [21], thus increasing serum calcium which results in sparing serum vitamin D. In addition, physical activity which is known to reduce body weight by increasing the rate of lipolysis may enhance mobilization from adipose tissue, thus increasing its serum level [22-25]. From the previous literature it appears that at least in adults, women have higher rates of noted induced systemic lipolysis than men [26]. This was not observed in the present study, suggesting that gender differences in lipolysis are not evident in children even in their adolescent stage. This needs further confirmation.

The lower vitamin D status levels observed in the cohort can be attributed to growth spurts of peak bone mass, requiring higher vitamin D and calcium nourishment, which is attained during pubertal skeletal growth [27-29]. Nevertheless, the high prevalence of vitamin D deficiency in the young Arab population living in a sunny-rich area is surprising and likely multi-factorial [30] including sun exposure, vitamin D, calcium intake [31,32], skin pigmentation, clothing, cultural practices [33], obesity and increasing age [16], and VDR polymorphisms [34].

Serum vitamin D levels were highest in the group exposed to sunlight on a daily basis as anticipated, but were nevertheless vitamin D deficient. Aside from the conventional factors previously mentioned contributing to vitamin D deficiency, the atmosphere in urban Riyadh is known to be saturated with dust particles and vehicle pollution that may affect the availability of UV radiation; since the dust is composed of mineral particles that both absorb and scatter sunlight and this might affect vitamin D synthesis [35].

Our study acknowledges a few limitations. Data on sun exposure and physical activity were based on administered questionnaires which are subject to recall bias. Data on which body parts were exposed were also not provided. A prospective approach using a more controlled environment (experimental setting) rather than interview questionnaire might provide more definitive answers to the differences observed in the present study. Lastly, serum parathormone was not assessed in the study and it is still debatable whether vitamin D supplementation should be given to those who are deficient in the absence of information on biological consequences and non-availability of serum parathormone level.

## Conclusion

In conclusion, vitamin D deficiency is very common among apparently healthy Saudi children and adolescents, as found in the present study. It is influenced by their level of physical activity and frequency of sun exposure. Increased outdoor physical activity among Saudi children and adolescents should be encouraged not only at home but also in schools to promote a more active life style that will counteract not only vitamin D deficiency but also conditions such as childhood obesity. Vitamin D supplementation in this vulnerable group is also suggested but needs further studies.

## Competing interests

The authors declare no conflict of interest, both financial and non-financial for this study.

## Authors’ contributions

AA, SA and NMA for conception and design; KMA, YA, OSA and MSA for the acquisition of subjects and interpretation of data; DSY and OM for the subject recruitment and sample collection; SK for the manuscript development; SS and GC for the intellectual input and the final version of the manuscript. All authors have seen and approved the final version of the paper.

## Pre-publication history

The pre-publication history for this paper can be accessed here:

http://www.biomedcentral.com/1471-2431/12/92/prepub
